# Can repetitive mechanical motion cause structural damage to axons?

**DOI:** 10.3389/fnmol.2024.1371738

**Published:** 2024-06-07

**Authors:** Allegra Coppini, Alessandro Falconieri, Oz Mualem, Syeda Rubaiya Nasrin, Marine Roudon, Gadiel Saper, Henry Hess, Akira Kakugo, Vittoria Raffa, Orit Shefi

**Affiliations:** ^1^Department of Biology, Università di Pisa, Pisa, Italy; ^2^Faculty of Engineering, Bar Ilan Institute of Nanotechnologies and Advanced Materials, Gonda Brain Research Center, Bar Ilan University, Ramat Gan, Israel; ^3^Graduate School of Science, Division of Physics and Astronomy, Kyoto University, Kyoto, Japan; ^4^Department of Biomedical Engineering, Columbia University, New York, NY, United States

**Keywords:** neuron, axon, cytoskeleton, mechanical fatigue, mechanobiology

## Abstract

Biological structures have evolved to very efficiently generate, transmit, and withstand mechanical forces. These biological examples have inspired mechanical engineers for centuries and led to the development of critical insights and concepts. However, progress in mechanical engineering also raises new questions about biological structures. The past decades have seen the increasing study of failure of engineered structures due to repetitive loading, and its origin in processes such as materials fatigue. Repetitive loading is also experienced by some neurons, for example in the peripheral nervous system. This perspective, after briefly introducing the engineering concept of mechanical fatigue, aims to discuss the potential effects based on our knowledge of cellular responses to mechanical stresses. A particular focus of our discussion are the effects of mechanical stress on axons and their cytoskeletal structures. Furthermore, we highlight the difficulty of imaging these structures and the promise of new microscopy techniques. The identification of repair mechanisms and paradigms underlying long-term stability is an exciting and emerging topic in biology as well as a potential source of inspiration for engineers.

## 1 Introduction

Biological structures have evolved to generate, transmit, and withstand mechanical forces in a highly sophisticated manner. Biological examples have inspired mechanical engineers for centuries and led to the development of critical insights and concepts. For example, “tensegrity” refers to the emergence of stability as a result of a balance of tensile and compressive elements in a mechanical structure. It was inspired by the human musculoskeletal system, applied to architectural designs, and returned to biology as a framework to understand cellular mechanics (Wang N. et al., 2001; Ingber et al., 2014).

However, progress in the engineering discipline of mechanics also raises new questions about biological structures. The past decades have seen the increasing study of failure of engineered structures, and its origin in processes such as materials fatigue. Now engineers ask not only “Which mechanical design elements enable a human to walk upright?” but also “What enables a human to walk upright for 100 years, given what we know about mechanical degradation”? At the tissue level, we now understand that some tissues, such as bone, are in a dynamic equilibrium between assembly and disassembly (Florencio-Silva et al., 2015) which extends their lifetime dramatically by enabling constant repair (Einhorn, 1998; Bates et al., 2018). Other tissues, such as the spinal cord, are formed from long-lived cells which maintain their function over the entire lifetime of the organism (Liddell, 1960). How do these cells maintain their operation despite being constantly subjected to mechanical stresses? How do the mechanical stresses degrade intracellular structures, and what mechanisms are activated to effect repair? These are the fundamental biological questions currently under investigation.

Of particular interest is the axon, because – in addition to transmitting information – it is a unique mechanical structure. Mechanical structures such as rods are the default structures in mechanical engineering used to illustrate how applied forces generate locally varying stresses. These stresses are translated into locally varying strains based on the local materials properties, which are then integrated into deformations. Repeated deformations can lead to failure at subcritical stresses due to materials fatigue (Suresh, 1998), as was discovered by Wöhler in the study of axles of rail cars (again slender rods) in the 19th century (Spangenberg, 1875). While the neuron is shaped like a long slender rod, it differs from rods by its ability to heal which makes it the perfect candidate to examine dynamic materials.

The effect of mechanical forces on neurons has of course been extensively studied in biophysics and cell biology, but primarily when a constant or transient load is applied (Karafyllidis and Lagoudas, 2007; Hamant et al., 2019; Thompson et al., 2019). These studies yielded, e.g., insights into the response of neurons to stretching in the course of traumatic brain injury (Farkas et al., 2006), or the effect of applied mechanical forces on axonal regeneration (Shibuya et al., 2009). The mechanical integrity of axons is not only challenged by external mechanical forces but also internal mechanical forces, including forces generated by motor protein and forces generated by microtubule polymerization (Padmanabhan and Goodhill, 2018; Hahn et al., 2019; Franze, 2020; Raffa, 2023). Microtubules not only play a role in stabilizing axons mechanically but also in axon functionality (Kapitein and Hoogenraad, 2015; Che et al., 2016; Kaplan et al., 2018; Kelliher et al., 2019). *In vivo*, they are essential for axon elongation, guidance and connectivity (Miller and Suter, 2018). They provide the cytoskeletal “tracks” for transportation of proteins, vesicles and granules (Namba et al., 2011; Maday et al., 2014). However, the functional response of microtubules in homeostasis and under repetitive mechanical stimulation remain largely unknown.

The increased stability of neuronal microtubules compared to other microtubules and the effect of drugs on it has been highlighted in the recent literature (Baas et al., 2016; Hamant et al., 2019). The effects of mechanical stress have been studied in the context of traumatic brain injury. Short-term large (>30%) stretching of axons has been found to lead to breaking of microtubules and subsequent axon degeneration (Tang-Schomer et al., 2010). Local complete photo-damage to microtubules has highlighted molecular mechanisms of repair (Aumeier et al., 2016). The mechanisms of repair and adaptation have not yet been elucidated. Recent efforts to model the damage and failure of microtubules using molecular dynamics simulations apply unrealistic strain rates due to computational time limitations (Manuchehrfar and Shamloo, 2018; Wu and Adnan, 2018). Hahn et al. have synthesized the existing knowledge about axon homeostasis and highlighted the complexity of axonal structure and regulation as well as the prominent role of microtubule bundles (Hahn et al., 2019).

Understanding the fundamental mechanobiology of axonal homeostasis is of potentially significant biomedical value, due to the proposed role of axon decay as key trigger for neuronal decay observed in aging and neurodegenerative disorders (Adalbert and Coleman, 2013; Salvadores et al., 2017; Jakobs and Franze, 2020). However, our primary interest is fundamental biology and its intersection with biomechanics at the molecular and nanoscale.

Here, we would like to aggregate the accumulating information about the mechanisms responsible for axonal homeostasis and develop a better appreciation of the cell as a “self-repairing machine.” The novelty of our discussion is rooted in that an engineering perspective is taken and applied to cell biology by asking not “How does it work?” or “How does it break?” but “How does it keep working for so long”? For example, the functioning of healthy axons and their response to acute damage (e.g., as a result of traumatic brain injury) has been extensively studied, but the response to everyday stresses is a frontier. Biological strategies to maintain homeostasis of mechanically stressed cellular structures must exist but are largely undiscovered.

Mechanical structures can be maintained in a functional state by a design which maximizes lifetime through the use of hard materials (e.g., steel or the enamel of teeth) or through frequent repair (e.g., tires or bone). Recent advances in nanotechnology have enabled the study of the mechanical properties of biological nanostructures such as cytoskeletal filaments (Duan et al., 2015) and have even employed these biological nanostructures in hybrid nanodevices (Hess and Ross, 2017; Saper and Hess, 2019). In these devices, the disruption of the biological nanostructures by mechanical stresses often limits the device lifetime (Hess and Dumont, 2011; Dumont et al., 2015; Keya et al., 2017; Saper and Hess, 2019). It has been a recent discovery that these biological nanostructures are self-healing and can reverse mechanical damage if supplied with new building blocks in a microfluidics device (Schaedel et al., 2015, 2019; Aumeier et al., 2016). Long-lasting nanodevices enabled by the incorporation of self-repair mechanisms represent a frontier in nanotechnology.

Cells are faced with a similar need to maintain their subcellular structures in a working state, sometimes for over a century (Boateng and Goldspink, 2008). Generally speaking, the attention of science is currently focused more on the mechanisms of cellular functions than the long-term maintenance of the structures involved in these mechanisms or on acute injuries. However, aging and disease are often associated first with a breakdown of the repair mechanisms before functional decay becomes observable. It is our goal to advance our understanding of the cellular repair mechanisms counteracting damage resulting from repeatedly applied mechanical stresses.

Exerting defined mechanical stresses on cells and their subcellular mechanical structure is generally challenging, but easier for the axons of neurons. The axon is a cellular structure to which we can adapt our existing tools developed for the investigation of the nanomechanics of microtubules (Kabir et al., 2012, 2015). Its growth pattern is affected by mechanical constraints (Shefi et al., 2004) and physical mechanical interactions (Baranes et al., 2012). The axon is also of particular relevance since its functioning is impacted by neurodegenerative diseases (Gunawardena et al., 2000), and lends itself to the controlled application of mechanical stress cycles and the observation of the effects, as well as to the modeling of the nanoscale mechanics of deformation.

Here, we aim to outline the mechanical stresses applied to axons, summarize how engineered structures respond to repeated mechanical stresses, discuss how controlled mechanical stresses can be applied repeatedly to biological structures, discuss how cytoskeletal structures respond to repeated stresses, and how failure events resulting from mechanical stresses may be imaged with modern microscopy techniques.

## 2 Mechanical motion in the nervous system

The impact of daily movement on the internal structure of nerves is unclear and few studies have been done to date. While some studies have investigated the effects of traumatic brain injury, others have analyzed cultured neurons in the absence of mechanical motion (Bray and Bunge, 1981; Jafari et al., 1997; Antman-Passig et al., 2017; Ju et al., 2017). However, the peripheral nervous system – unlike the central nervous system – is subjected to constant movements within the joints and could be affected by mechanical fatigue, which is damage caused by repeated loading with fluctuating stresses and strains. The nerves, and therefore the axon bundles that are part of them, are regularly exposed to three types of mechanical stress: tension, compression and bending (Topp and Boyd, 2006). For example, in the human arm, the median and ulnar nerves are subjected to one of the most common movements in daily life, cycles of flexion and extension at different arm joints. Bending an elbow by 90° results in a radius of curvature of approximately 4 cm for the median nerve and 3 cm for the ulnar nerve. In the human body, nerves in the resting position are stretched by 10% (Topp and Boyd, 2006), while daily movements can increase the strain up to 14% (Wright et al., 2001). Force measurements of flexion and extension movements of the elbow or wrist performed on embalmed bodies (allowing for direct measurements) using a buckle force transducer indicated a 2 to 4% stretching (Byl et al., 2002). Furthermore, while the nerve is elongated, there is a reduction in the cross-section, which leads to lateral compression. Forces on the nerves are reduced by excursion, in which the peripheral nerves slide or glide relative to the surrounding tissue mainly in the joint area (Topp and Boyd, 2012). Tension and compression stresses have not shown any effect on the axons over a low number of cycles. Studies agree that the viscoelastic properties of nerves allow them to cope with the mechanical stresses they experience on a daily basis (Phillips et al., 2004). The strain-limit value, the value below which there is no damage on the internal structures, is not reached during daily stretching and compressing movement (Dennerll et al., 1989). However, these results were obtained from studies on cadavers or static cultured neurons. The number of flexion and extension cycles sustained by the axons of the median and ulnar nerves, assuming a human lifetime of 80 years, movement for 16 h a day, and 5 flexion and extension movements per minute, is estimated to be approximately 100 million. Human median and ulnar nerves, therefore, sustain over their lifetime approximately 100 million extension and flexion cycles with a strain of 2–4% and a radius of curvature of 3–4 cm. The peripheral nerves of smaller animals experience a roughly similar number of cycles since a shorter lifespan is balanced by more frequent movement, similar strain levels due to geometric similarity and smaller radii of curvature (in proportion to linear size). Many engineered structures, such as electric cables, would not perform reliably when subjected to mechanical stresses of this magnitude, as we will discuss in the next section.

## 3 Mechanical fatigue in engineered and biological materials

**Figure 1:**
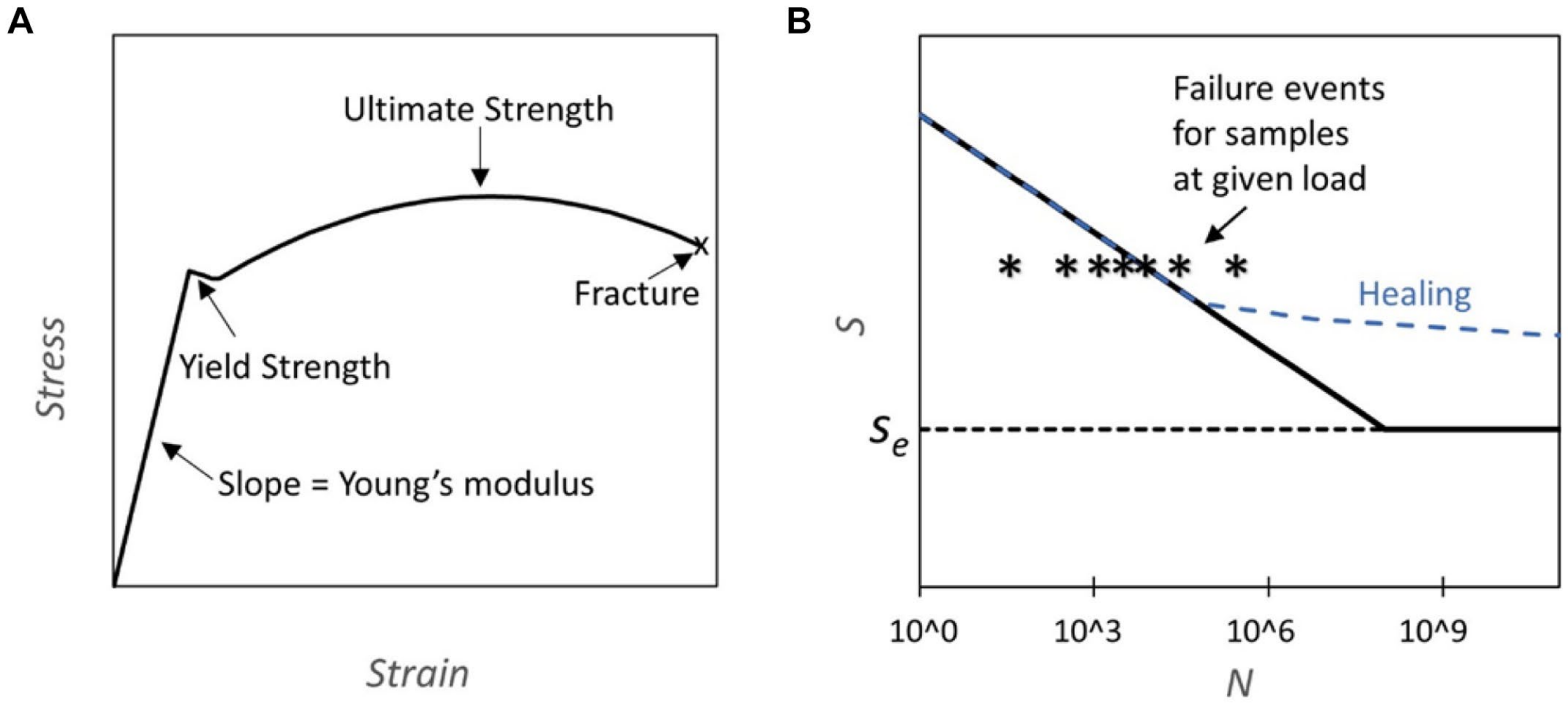
**(A)** Stress strain curve: the plot represents an example of a stress strain curve typical for steel where different properties of the material can be assessed (Yun and Gardner, 2017). **(B)** S-N Diagrams and Healing Capability: This figure presents S-N diagrams, where S represents fatigue strength and N the number of cycles. The diagrams are constructed through subjecting components to cycles of predetermined load until failure. The failure line (solid black) on the graph indicates the cycle count at which 50% of the tested samples fail under a specific load. Notably, some materials exhibit an endurance limit (S_e_), denoting a threshold below which fatigue failure does not occur. Conversely, other materials eventually experience fatigue failure regardless of the applied load. The dashed blue line illustrates how the curve may be altered due to healing, which is activated during normal usage.

The application of mechanical stress to materials can lead to deformation and, ultimately, fracture or failure. The progression from elastic deformation over yielding to eventual fracture under uniaxial load conditions is depicted in the stress–strain curve (Figure 1A) (Courtney, 2005). Repeated application of stress over thousands or millions of cycles can lead to failure at loads significantly below the yield stress due to the formation and growth of cracks. This phenomenon is termed mechanical fatigue (Suresh, 1998). Mechanical fatigue assumes a pivotal role in evaluating the endurance and dependability of structural components subjected to the cyclic loading conditions often present in aerospace, automotive, and civil engineering. The fatigue behavior of a material is often captured in a SN plot, where the number of cycles to failure N at a given stress level is shown on the abscissa and the corresponding stress level on the ordinate. (Figure 1B). For some engineering materials cyclic stresses below a stress level termed “endurance limit” do not ever lead to fracture and permit an infinite lifetime.

For example, the Ti Al6 V4 titanium alloy stands out for its durability, tolerating 10^4^ cycles at 700 MPa and a practically infinite number of cycles below its endurance limit of 600 MPa (Fleck and Eifler, 2010). For comparison, cold-rolled mild steel can endure 300 MPa over 10^6^ cycles and exhibits an endurance limit of 270 MPa (Forrest, 2013). In biomechanics, fatigue testing has been conducted for bones which are cyclically loaded during walking and running (Taylor, 1998; Acevedo et al., 2018; Edwards, 2018). Bone fatigue arises from microcracks within the bone matrix that may undergo partial repair before the repair cycle is disrupted, leading to crack propagation. This phenomenon is accentuated in older individuals due to a decline in bone density.

Mechanical fatigue is also experienced by biological materials (Arola et al., 2010; Edwards, 2018; Qiang et al., 2019). For example, Nasrin et al. conducted fatigue measurements on microtubules and extrapolated that microtubules can withstand 10^3^ cycles of 20% compression and 5 × 10^6^ cycles of 12.5% compression. However, further experiments are required to ascertain the existence of an endurance limit for microtubules (Nasrin et al., 2023). Unlike inanimate materials, which often incur irreparable damage from fractures or fatigue, living organisms possess an exceptional capability to self-heal. When a biological material begins to accumulate damage, it can engage in a regenerative process whereby the compromised region is renewed and fortified with fresh components. This process can occur at multiple scales, from the molecular to the tissue level. At the molecular scale for example, microtubules can undergo replenishment through the replacement or addition of new tubulin building blocks (Ganser and Uchihashi, 2019), and biomolecular motors in muscles are systematically replaced (Boateng and Goldspink, 2008). In instances where the healing process is impaired or absent, the biological material becomes more susceptible to fracture (Schaedel et al., 2015). An illustration of this is that bones experiencing a physiological stress level are estimated to fracture during their lifetime, thus necessitating repair to avert failure (Taylor, 1998). This inherent mechanism serves as a safeguard, shielding the material from the accumulation of damage, as it perpetually renews and rehabilitates itself. This cycle of rejuvenation ensures the resilience and functionality of biological structures over time, presenting a potent countermeasure against the mechanical fatigue associated with non-living materials.

## 4 Application of repetitive mechanical stresses to cells

Neurons, heart, lungs, and muscles of a mammal are constantly under tensile-compressive stress cycles throughout development, growth, and adult life. Mechanical stress can trigger biological responses and promote biological processes like embryogenesis (Fruleux et al., 2019), development (Lecuit and Lenne, 2007; Le et al., 2016), and tissue homeostasis (Matthews et al., 2006; Barnes et al., 2017). The response of cells has been investigated by applying mechanical stresses in different modes, constant, transient, and repetitive.

Constant applied stress exerts a continuous force on cells modulating cellular structure and function. For instance, continuous tensile stress on endothelial cells alters cytoskeletal organization and focal adhesion dynamics (Bao and Suresh, 2003).

Mechanical stress suppresses the growth and proliferation of cardiac stem cells (Kurazumi et al., 2011) and human parametrial ligament fibroblasts (Hu et al., 2017) led by cytoskeletal depolymerization and rearrangement, indicating damage to F-actin in cells. Additionally, research demonstrated the impact of constant stress on neuronal cells, showing alterations in synaptic plasticity and neurotransmitter release dynamics (Uchida et al., 2014). Constant stress can lead to cell stiffening through tensegrity mechanisms, with structural remodeling occurring over time to solidify the cytoskeleton. Steady loading generally results in stiffening rather than softening of cells (Walker et al., 2020; Putra et al., 2023).

Transient stresses introduce temporary variations in mechanical load on cells. Over longer timescales, transient stretching of cells induces structural disorder within the cytoskeleton, causing a loss of stability and softening (Stamenović and Wang, 2011). A quantitative cell-based model suggested that cells adapt their volume with a certain delay in response to pressure changes due to internal friction and cytoskeletal remodeling (Van Liedekerke et al., 2019). Stretching under mechanical stress generates internal pressure to deform the cell membrane and contribute to volume changes in cells. This membrane stress can partially compensate for osmotic gradients and help maintain cell volume (Khmelinskii and Makarov, 2020). Cancer cells showed increased plasticity in behavior due to increasing stress (Onal et al., 2023).

Cyclic stress is the most pronounced mode of stress in living beings that imposes rhythmic mechanical cues on cells, triggering adaptive responses and physiological changes. The effects of cyclic stress on cellular behavior have provided insights into mechanotransduction mechanisms and biomechanical regulation by modulating cell adhesion and migration processes through alterations in cellular orientation and alignment. When cyclic stress is exerted in a single direction (uniaxially), causing deformation or strain along that specific axis, cells align perpendicular to the stress. In contrast, under equibiaxial stress, where stress is simultaneously applied along two balanced axes with equal intensity, resulting in uniform deformation or strain across both axes, cells show no clear orientation (Wang J. H. C. et al., 2001; Pennisi et al., 2011; Tondon and Kaunas, 2014). Mechanical stress applied to human fibroblasts leads to changes in the number, length, and area of vinculin-positive focal adhesion contacts. This is related to the roles of Akt and RhoA in focal adhesion assembly and maturation (Boccafoschi et al., 2010). The cardiac fibroblasts respond to mechanical stress by revealing changes in gene expression profiles associated with extracellular matrix remodeling and tissue homeostasis (Jungbauer et al., 2008; Greiner et al., 2013). A similar effect of cyclic stress is observed on vascular smooth muscle cells (Liu et al., 2008) and osteosarcoma cells (Alloisio et al., 2023), where the cell morphology was changed to a larger cell area and more elongated shape and expression of genes related to proliferation was increased two to three fold under stress. The frequency of cyclic stress and cell proliferation are negatively correlated (Duveau et al., 2024).

In summary, mechanical stress has diverse effects on different cell types, including reduced proliferation, cytoskeletal changes, altered adhesion and signaling, and changes in membrane mechanics and cell volume regulation. The specific responses depend on the mode of the mechanical stress being applied. The cytoskeleton is the primary means of sensing, integrating, and coordinating cellular responses to mechanical stimuli and structural cues (McCain and Parker, 2011; Table 1).

**Table 1:** Effect of repetitive mechanical stress on different cell types.

Mechanical stress	Cell type	Cell function	Summary of the effect	Reference
5 and 10% uniaxial stretching at 0.5 and 0.25 Hz for upto 3 h	human aortic endothelial cell	Cell alignment	Cells align perpendicularly to strain direction	Wang et al. (2001a)
5 and 10% equi-biaxial stretching at 0.5 and 0.25 Hz for upto 3 h	human aortic endothelial cells	Cell orientation	Cells remain randomly oriented	Wang et al. (2001a)
1 to 15% stretch at 0.0001 to 20 Hz for 1 to 5 h	human fibroblast	Cell alignment	Cells reorient perpendicular to the strain direction at two different rates separated by a threshold strain frequency	Jungbauer et al. (2008)
10% uniaxial stretching at 0.5, 1.0, 1.5, and 2.0 Hz for 24 h	human aortic smooth muscle cells	Cell alignment	Cells align perpendicularly to the stress direction	Liu et al. (2008)
2% uniaxial strain at 1 Hz for 3 h	human fibroblast	Cell adhesion	Stress disassembles focal contacts	Boccafoschi et al. (2010)
4.9, 8.4, 11.8, 14 and 32% uniaxial strain at 9 to 52 mHz depending for 16 h	human fibroblast	Cell orientation	Cells reorient independent of the amplitude and frequency of strain	Faust et al. (2011)
15% uniaxial stretching at 0.5 Hz for 48 h	mouse skeletal muscle myoblasts	Cell morphology and cell orientation	Cells are elongated and align perpendicularly to strain direction	Pennisi et al. (2011)
15% uniaxial stretching at 0.5 Hz for 48 h	mouse skeletal muscle myoblasts	Cell morphology and cell orientation	Cells are elongated and remain randomly oriented	Pennisi et al. (2011)
120% stretch at 1 Hz for 24 h	human cardiac stem cell	Cell proliferation	Cell proliferation is inhibited	Kurazumi et al. (2011)
120% stretch at 1 Hz for 24 h	human cardiac stem cell	Cell apoptosis	Stress increases apoptosis	Kurazumi et al. (2011)
8% uniaxial strain at 3 Hz upto 3 h	mouse embryonic *fibroblast*	Cell spreading	Cell spreading is perpendicular and polarized	Greiner et al. (2013)
10% uniaxial strain at 0.5 Hz upto 48 h	mouse spinal cord cell	Cell apoptosis	Stress induces apoptotic cell death	Uchida et al. (2014)
10% uniaxial stretch at 1 Hz for 3 h	human osteosarcoma	Cell alignment	Cell alignment is perpendicular on soft collagen gels and parallel on stiff silicone rubber sheets	Tondon and Kaunas (2014)
9 and 36% uniaxial strain at 0.1 Hz for 4 h	human fibroblast	Cell viability	Stress decreases cell viability	Hu et al. (2017)
0.5% uniaxial stretching at 1 Hz for 24 h	human osteosarcoma	Cell morphology	Cell area is enlarged and cell shape is elongated	Alloisio et al. (2023)

## 5 Effects of repetitive stress on cytoskeletal structures

**Figure 2:**
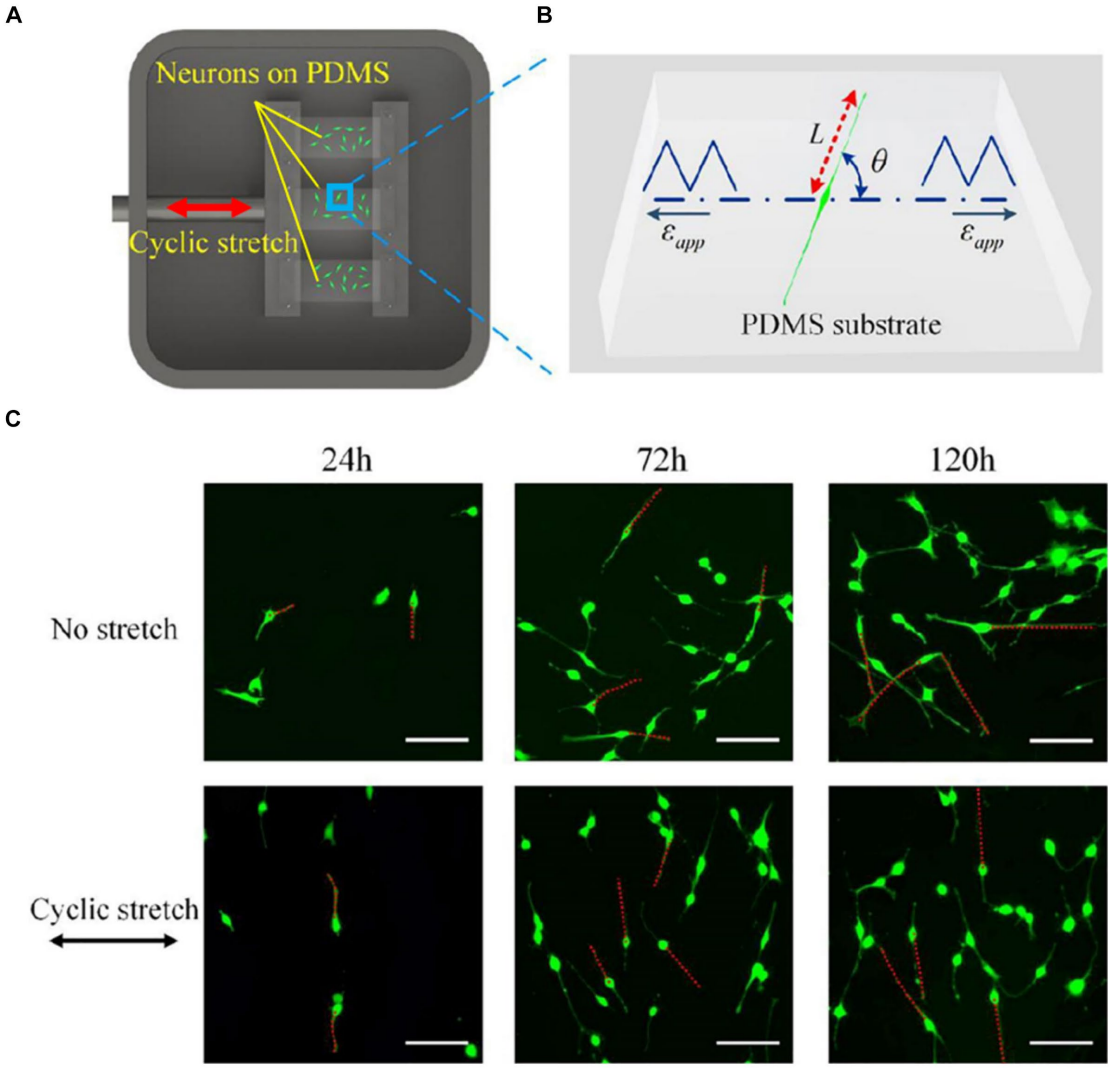
**(A)** A Scheme of an experimental setup for applying cyclic stretch on neurons. One end of an elastic polydimethylsiloxane (PDMS) film is attached to a fixed clamp and the other end is attached to a movable clamp. The PDMS is cyclically elongated. The direction of the elongation is indicated by the red arrow. **(B)** Snapshot of a PC12 cell subjected to cyclic stretch, where ε_app_ is the cyclic stretch, L is the length of the axon, and θ is the angle between axon alignment and stretch direction. **(C)** Morphological images of PC12 cells after 24, 72, and 120 h with and without applying cyclic stretching. Images in the upper row are obtained under no stretch and in the lower row are obtained under cyclic stretch (10% amplitude and 0.25 Hz frequency). Scale bar: 100 μm. Figure adapted from Lin et al. (2020).

Biological processes indispensably require responses to external mechanical stress (Hamant and Haswell, 2017; Sadhanasatish et al., 2023). Stress applied to a cell is distributed broadly by its cytoskeleton, but the magnitude of transmitted stress to a particular location depends on network mechanics and architecture and can have marked effects on cellular processes, from individual filament polymerization up to entire network reorganization (Fletcher and Mullins, 2010). When repetitive stretching is applied, cellular reorientation occurs in an alignment perpendicular to the direction of the stretching, as shown in Figure 2 (Liu et al., 2008; Faust et al., 2011; Sakamoto et al., 2017; Lin et al., 2020). Such cellular reorientation is assisted by the reorganization of the contacts of the cells to the extracellular matrix (ECM). Cells maintain a set point in the ECM and reorganize their stress fibers, adhesions, and traction forces (Saez et al., 2005). The cytoskeletal components contribute to the direct communication between cells and their ECMs by mediating integrins, associated with actin through formation of regulatory molecules (Verma et al., 2012). The repetitive application of stress causes progressive adaptation in cytoskeletal structures, leading to reversible compression in proteins like α-actinin (Verma et al., 2012). This adaptation occurs over multiple time scales, involving rapid and reversible changes in cytoskeletal stresses followed by chronic rearrangements. Myotubes, when subjected to repetitive stretching, exhibited increased synthesis of myosin compared to unstimulated conditions (Vandenburgh and Kaufman, 1979).

In response to repetitive mechanical stress, the cytoskeleton exhibits dynamic responses characterized by changes in stiffness, self-repair mechanisms and enhanced stabilization. Microtubules soften under mechanical stress, with repeated bending cycles causing incremental softening and material fatigue. The maximal deflection of microtubules increases with each mechanical cycle, indicating a progressive softening effect under repeated stress (Schaedel et al., 2015). As response to cycles of compressive forces in living cells, microtubules become distorted, less dynamic and more stable (Li et al., 2023). Actin filaments responds to cyclic stretch through reversible stress softening (Chaudhuri et al., 2007). Actin depolymerization and repolymerization play a significant role in strain softening and recovery responses under mechanical stress (Walker et al., 2020).

## 6 Effects of mechanical stress on neurons

**Figure 3:**
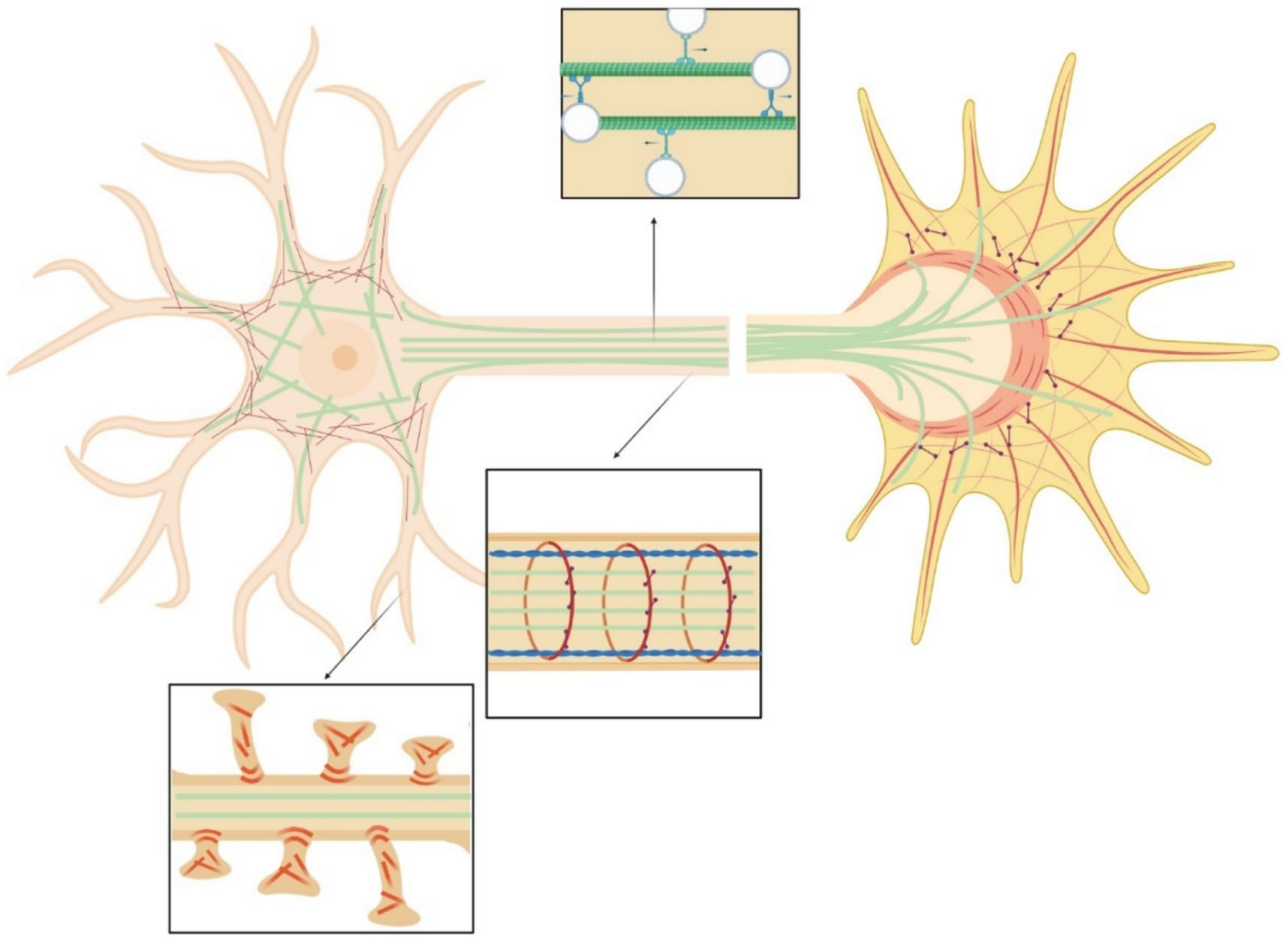
Scheme of the neural cytoskeleton: actin fibers (red), microtubules (green), myosin (purple), spectrin (blue). Top insert highlights vesicles transport in the axon. Central insert illustrates actin-spectrin rings. Bottom inset shows dendritic spines.

**Figure 4:**
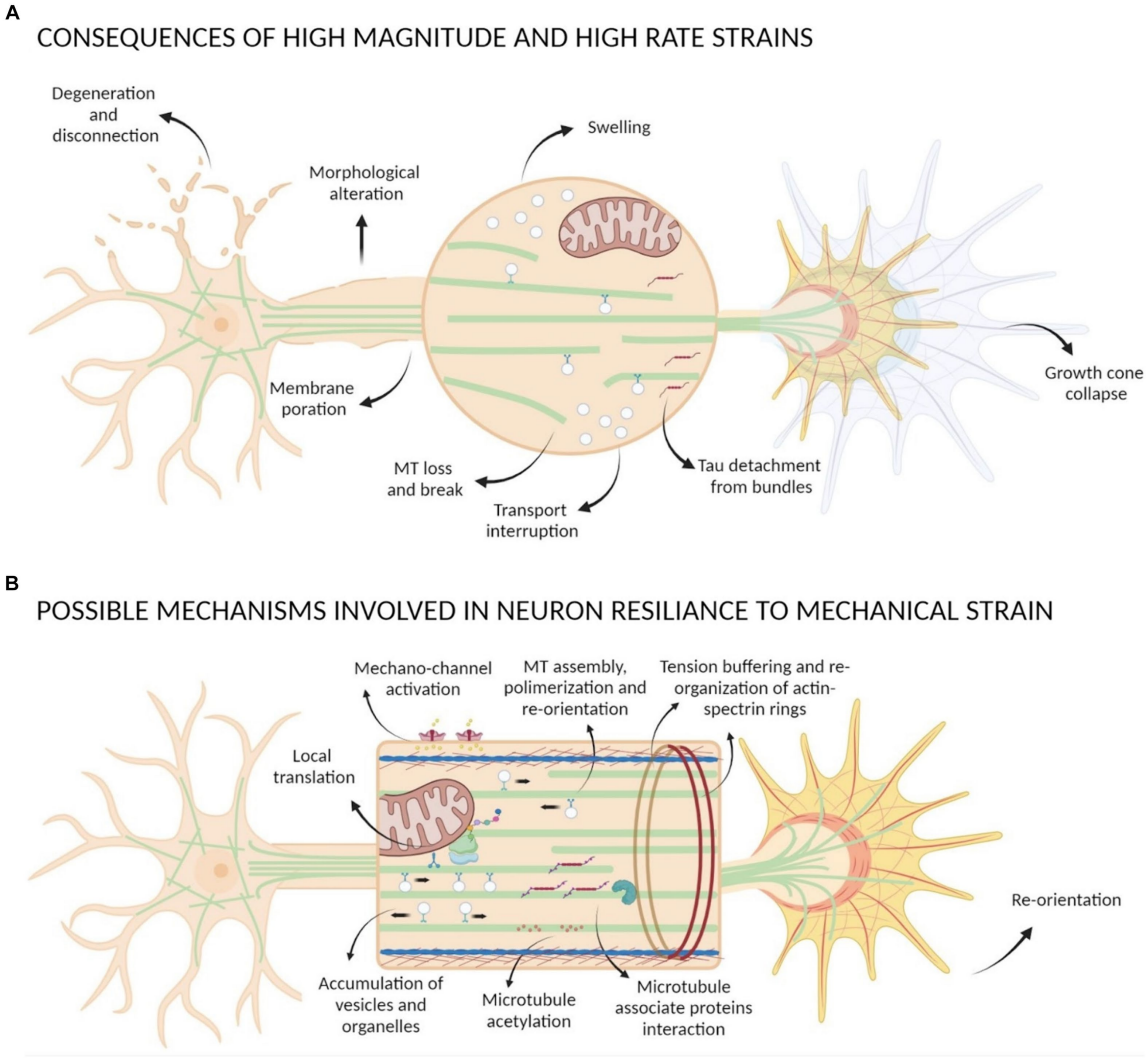
Scheme of mechanical strain consequences on neurons. **(A)** Strains with high magnitude and high rate can cause morphological alteration of neurons, degeneration and disconnection, membrane poration, GC collapse, Tau detachment from bundles, loss and break of MTs causing transport interruption and formation of swelling. **(B)** Possible mechanisms involved in neuron resilience to mechanical strain comprehend activation of mechano-sensible channels, GC re-orientation, tension buffering mediated by actin-spectrin rings re-organization, MTs assembly and polymerization that lead to an accumulation of vesicles and organelles with local translation events, interactions between MAPs and MTs and post-translational modification of tubulin such as acetylation.

Neurons, as all cells, interact physically with the surrounding environment and through a process known as mechanotransduction perceive the mechanical stimuli and convert them into a biochemical response (Watson, 1991; French, 1992). Through this process they adapt to and survive in a changing environment. Neurons experience mechanical stimuli during neurodevelopment, aging, pathologic conditions and in everyday function, homeostatic process, and movement (Butler, 1989; Sarkis et al., 2017; Chighizola et al., 2019; Javier-Torrent et al., 2021). Neuronal mechanical interactions affect their expression regulation (Baranes et al., 2019). However, the signaling cascade of mechanotransduction in neurons is still only partially discovered. The repeated observations that mechanical strains can induce a cytoskeletal remodeling inside cells suggest that the cytoskeleton could be strongly involved in the transduction process. Exogenous mechanical stimuli are perceived by neurons and propagated within the cell through a complex network that spans from the cell membrane to the nuclear membrane (Figure 3). The interaction with matrix is mediated by cell adhesion molecules of the immunoglobulin superfamily (IgSF), including NCAM and L1 family members (Leshchyns'ka and Sytnyk, 2016). Their extracellular domain, containing one or several immunoglobulin-like (Ig) repeats typically mediate interactions with proteins of the extracellular matrix (ECM) (Grumet and Sakurai, 1996). The intracellular domains of IgSF cell adhesion molecules (CAMs) interact with the components of the cytoskeleton including the submembrane actin-spectrin meshwork, actin microfilaments, and microtubules. Similarly to non-neuronal adhesion points, IgSF CAMs can trigger the recruitment of multiple scaffold proteins thereby amplifying the interactions with the cytoskeleton, leading to the maturation the point contact (PC) adhesion and promotion of intracellular force generation by actomyosin contraction. Forces generated intracellularly by the actin cytoskeleton can be transmitted to the cell cytoskeleton via physical coupling between the F-actin filaments and the microtubules through a number of common binding proteins and regulators (Dogterom and Koenderink, 2019). Interestingly, the cell cytoskeleton is also coupled to the nucleoskeleton through the LINC (LInkers of the Nucleoskeleton to the Cytoskeleton) complex formed by the SUN and KASH protein domains. SUN proteins cross the inner nuclear membrane (INM), enabling their N-termini to bind to the nuclear lamina, while KASH domain proteins cross the outer nuclear membrane (ONM), enabling their N-termini to bind to the cytoskeleton. Similarly to the actin stress fibers anchored by the PC adhesions that are involved in ECM / cytockeleton transmission of contractile forces through actin-myosin dynamics, it has been reported that an actin cap, composed by bundles of highly contractile acto-myosin filaments anchored to the apical surface of the interphase nucleus, is involved in force transmission from cytoskeleton to nucleoskeleton (Kim et al., 2013). Through this network, mechanical forces can also propagate from cell periphery to the nucleus (Li et al., 2014). Ingber et al. proposed a “tensegrity” model in which the cytoskeleton supports the tension inside cells by maintaining a balance between the forces at play. Dynamic cytoskeletal elements, like the contractile actomyosin and the compression-bearing microtubules, would participate in the propagation and diffusion of the mechanical stresses across the cytoskeleton of the cell (Ingber et al., 1981; Ingber, 1993; Wang et al., 1993; Ingber, 1997; Brangwynne et al., 2006; Figure 4). Even if myosin and actin have been first proposed to be involved in mechanosensing, now many articles are identifying microtubules as important players in force transduction and sensing events (Falconieri et al., 2023a).

The forces that are experienced by neurons can be divided into internal forces, determined mainly by re-adjustments of the cytoskeleton in response to external stimuli, and exogenous forces, which are the focus of this review. The exogenous forces can be divided into forces of extension or stretching, compression, and bending.

### 6.1 Extension/stretching

The effects of stretching are the most investigated; they have been studied since the 1940s in relation to body growth, neurodevelopment, and axon elongation (Weiss, 1941; Bray, 1984; Van Essen, 1997). Nowadays, it is known that low stretching forces (from very low pN to a few nN) can modulate a plethora of events like axon elongation, branching, axon excitability, transport alteration, orientation, and cytoskeletal dynamics (De Vincentiis et al., 2020b). Several thresholds for tensile forces have been identified that discriminate between elongation or axon breakage / growth cone (GC) collapse. For example, a threshold of sensitivity to mechanical strain required to promote neurite initiation was identified, and it was observed that the sensitivity changed with the neuronal type: a force of 0.5–1.5 nN was required in chick sensory neurons, 3–10 nN in PC12 neurons, and 0.31 ± 0.06 nN in chick forebrain neurons (Zheng et al., 1991; Chada et al., 1997; Lamoureux et al., 1997). Furthermore, axons cannot tolerate forces that exceed a maximum limit: Smith and colleagues found that axons of neurons could tolerate a 1 mm/day stretch rate, but when the stretch rate was doubled axons disconnected and were unable to sustain the elongation (Smith et al., 2001); Franze and colleagues determined that strains above 274 pN/mm^2^ caused GC collapse, neurite retraction and growth in another direction (Franze et al., 2009). Interestingly, Pfister and colleagues showed that the use of an acclimatation time could increase the threshold of tolerance for DRG neurons to stretching above which axons underwent disconnection (Pfister et al., 2004).

The effect of mechanical strain is not only related to the magnitude but also to the strain direction and the rate of application. Uniaxial strain on axons seem to be most damaging when the applied strain is parallel to the axons rather than inclined (Nakadate et al., 2014, 2017). Moreover, axons can tolerate high extension (until twice their original length) if the applied strain rate is low, but if the strain is applied quickly (< 50 ms) consequences are more drastic (Tang-Schomer et al., 2010); for instance, uniaxial strain above 65% applied with a 26–35 1/s strain rate has been found to cause primary axotomy (Smith et al., 1999). When the insult is above the threshold, cells cannot put in place counteracting mechanisms due to a failure of their structures. Tang-Shomer and colleagues have shown that application of high dynamic strains on cortical neurons causes distortions and axon swelling along axons after they relax back within the first hours after stimulation, and degeneration in most axons within 24 h. They hypothesize that the appearance of varicosities along axons is due to transport interruption, as they observed that breakage, buckling and reduction of microtubules take place immediately after the injury (Tang-Schomer et al., 2010, 2012). The sensibility of microtubules to high strain rates has been also highlighted by *in vitro* studies showing how the high rate can cause microtubules breaking and detachment from bundles due to the breaking of tau-tau bonds (Ahmadzadeh et al., 2014, 2015).

Interestingly, a comprehensive analysis of the works published in the last four decades shows that the elongation rate per applied force calculated in previous studies is surprisingly similar (0.1–1 μm/h/pN) (De Vincentiis et al., 2020a), irrespective of the force magnitude (from 1 pN to 100 nN) and the model. The interpretation of this fact is that when axons are chronically stimulated with low forces, similarly to those generated physiologically (generally below 500 pN) (De Vincentiis et al., 2020b), they can sustain the stress over days to weeks, promoting axon elongation. When axons are stimulated acutely with high intensity forces, e.g., those generated in traumatic events (>1 nN), the axons thin and break in minutes if a resting time is not allowed to transport or neo-synthesize the mass necessary to counteract the applied strain.

Neurite elongation induced by mechanical stimulation can be productive, and it has been highlighted that MTs assembly and polymerization may be involved (Zheng et al., 1993; Putnam et al., 2001; Lee and Suter, 2008; Miller and Suter, 2018; Raffa et al., 2018; De Vincentiis et al., 2020a; Falconieri et al., 2022, 2023b). It has been proposed that mechanical signals could influence MTs stability by acting on the MT lattice structure (Gudimchuk et al., 2020), by a modulation of MAP function (Franck et al., 2007; Trushko et al., 2013), or by post-translational modification of tubulin for review see (Kerr et al., 2015; Robison et al., 2016; Swiatlowska et al., 2020; Coleman et al., 2021; Torrino et al., 2021; Seetharaman et al., 2022; Falconieri et al., 2023a). Recently, our group demonstrated a connection between axonal elongation and the modulation of MT dynamics in hippocampal neurons. Specifically, we found that forces generated by magnetic nanoparticles promote axonal elongation which was accompanied by an increase in microtubule stability and density (De Vincentiis et al., 2020a; Falconieri et al., 2022, 2023b). Stable MTs present a different surface that is recognized by motor proteins or MAPs, remaining stably bound to long-lived MTs (Jansen et al., 2021). We proposed that the addition of mass induced by force application is caused by this increase in microtubule stabilization/density that results in accumulation of organelles and vesicles in the axon, which, in turn, increases the probability of local translational events. This increase in axonal transport and the activation of local translation can provide the necessary mass to sustain axon elongation and neuronal maturation.

### 6.2 Compression

In contrast to stretching forces, the mechanotransduction of compressive strain is investigated less in neurons. Interesting studies in this field have been conducted on plant cells. These works have shown how microtubules can undergo a re-orientation during anisotropic wall stress or constrain and microindentation of apical meristems (Williamson, 1990; Hejnowicz et al., 2000; Hamant et al., 2008; Louveaux et al., 2016). In neural cells, many studies have been done in relation to traumatic brain injuries and spinal cord injuries, where the compressive force has been applied in-plane or perpendicular to the plane of the tissue. Similarly to the threshold behavior observed for tensile strain, a threshold has to be exceeded for compression to generate a response by cells, while overcoming a maximum limit induces cell damage and death. The application of a low compression force (<55 kPa), perpendicular to the axon plane, showed in hippocampal neurons that most axons are still able to grow after the injury, but when the applied stress rises to 55 kPa the percentage of growing axons decreased to 8% with swelling phenomena and thinning of the axonal membrane, while severe stress (>95 kPa) caused an immediate transection of the axon (Hosmane et al., 2011). By using a similar approach on axons of hippocampal neurons, Fournier and colleagues showed that a vertical focal compression prompted axon swelling and changes in cytoskeletal distribution. More in detail, they observed a decrease in neurofilament and MT density, a decrease in their number and MT break point and disorganization (Fournier et al., 2015). Compressive stresses higher than 0.5 MPa have been seen to strongly impact neuron viability. Most of these studies rely on instruments based on compressed gas to apply a vertical pressure, in the range of 0.5 to 1 MPa, to neurons. In these cases, data show a decrease of cell viability and an increase of apoptotic processes (magnitude and time dependent), oxidative stress, an increase of intracellular calcium, mitochondrial dysfunction, and ER stress (Ye et al., 2012; Quan et al., 2014; Chen et al., 2019). Similarly, to high stretching strain, these high compressive strains have been found to impact the cytoskeleton leading to its remodeling and events like microtubules disruption, disorganization and degeneration (Ye et al., 2012; Quan et al., 2014).

Studies conducted by application of in-plane compressions have instead alighted other interesting consequences on the cytoskeleton. For instance, by cell membrane compression, microtubule buckling has been observed in beating cardiac myocytes, cells compressed with a glass microneedle, and in cells in the proximal region of the cell membrane (Wang N. et al., 2001; Brangwynne et al., 2006). A recent study on retinal pigmental epithelial cells has shown that a compressive strain of 40% induces cell detachment, but when the strain is decreased to 10% there is no impact on cell shape; however, with the same strain, 1 Hz rate causes cell detachment while 0.1 Hz is well tolerated. In the same work, authors report a reduction of microtubule growth and an increase of microtubule stability through the relocation of plus-tip proteins (EB1 and CLASP2) from the microtubule end to the microtubule shaft, suggesting their central role in mechano-response (Li et al., 2023). Although these studies focused on non-neuronal cells, they offer insights into how compression frequency and force impacts cells, potentially affecting neurons in a similar way.

### 6.3 Bending/shear stress

Neurons can be subjected to a bending load through body movements, or the shear stress generated by body fluids. One of the models used to study the effect of axon bending on signal transduction pathways is *C. elegans*. The motion of *C. elegans* consists in a dorso-ventral bending by which the animal can move through a process modulated by proprioception (Goulding, 2012; Yeon et al., 2018). DVA, PMD, SMD proprioceptive neurons and the motor neurons can be activated by body bending and mechanosensitive channels could be involved in their activation and in the signal transduction (Li et al., 2006; Wen et al., 2012; Schafer, 2015; Yeon et al., 2018; Das et al., 2019, 2021; Tao et al., 2019; Liu et al., 2020). In DVA neurons, for instance, an increase of calcium level takes place following body bending, and the neuron activation seems to be dependent on TRP4 channels (Li et al., 2006). In SMDD neurons, in contrast, trp1 and trp2 channels have been found activated during body movement (Yeon et al., 2018). Similarly, in Drosophila nociceptors have been found activated by shear stress through TrpA1 (Gong et al., 2022).

Studies on plants models suggest that microtubules also may have a key role in signal mechanotransduction induced by a bending load (Nick, 2008). For instance, when the epidermis of maize coleoptiles is subjected to bending, MTs re-orient acquiring a longitudinal and transversal orientation at the inner (compressed) and outer (extended) side (Zandomeni and Schopfer, 1994; Fischer and Schopfer, 1997). Another study, on Azuki epicotyl, showed that microtubule orientation was altered by an inhibitor of stretch activated channels suggesting that mechanosensitive channels and microtubules could be strongly involved in this process (Ikushima and Shimmen, 2005).

The direction of axonal growth was found to change in response to the application of a pico-Newton shear stress. Growth cones of individual axons turn in response to a shear force of 0.17 pN generated by the controlled rotation of an optically driven particle (Wu et al., 2012). Similarly, neurites were found to preferentially align to the direction of the applied force, when a tangential force of a few pN was generated on MNP-loaded neurites through magnetic fields (Riggio et al., 2014). However, studies about shear stress in neurons have highlighted the presence of a threshold of tolerability. Kilinc and coworkers designed a microfluidic device to generate hydrodynamic shear stress (Blackman et al., 2000), observing that a fluid shear stress above 6 μPa caused the detachment of primary chick forebrain neurons, whereas at 4.5 μPa shear stress beading events took place but cells survived (Kilinc et al., 2008). Shear stress caused mechanical damage to the axolemma (axolemmal pores), calcium influx, Calpain activity, disruption of the cytoskeleton, and accumulation of mitochondria in the points of axonal bending, suggesting a causal relationship between membrane damage, Ca^2+^ influx, calpain dynamics, microtubule breaking and the formation of axonal beads (Kilinc et al., 2008, 2009). A microfluidic device was used to generate a cyclic shear stress to investigate how the stress repetition could impact on cells. In presence of high bending load, polymerizing tubulin was reduced together with an increase of cell elasticity; this observation suggests that the loss of microtubules could be related to a reduced resistance to deformation. From these findings, authors speculated that the cytoskeleton and the time for it to reassemble could play a crucial role in the protection from injury (Edwards et al., 2001). Taken together these studies suggest that shear stress can influence axonal growth at low levels, but high or repetitive stress can damage neurons through membrane disruption and cytoskeletal impairment.

All these insights show that neural mechanotransduction is a complex mechanism in which many factors are involved. A high strain magnitude and fast rate of application can severely affect neurons, even if neurons tolerate repeated strain with a resting time in between so that counteracting mechanisms can be put in place (Pfister et al., 2004). Cells cannot repair the damage if the applied strains are above a threshold, because of failure in cellular structure and function, as microtubules break and axonal transport interruption. Which homeostatic mechanisms help cell resilience at lower strain is still unclear, however, here we discussed some examples in which microtubules, their posttranslational modifications and associated proteins seem to be involved. Long-lived microtubules are acetylated on lysine 40 of α-tubulin (αK40) inside their lumen. Microtubules of neurons that are submitted to repetitive mechanical stress are highly acetylated (Janke and Montagnac, 2017). It was reported that αK40 acetylation modifies the microtubule lattice to better adapt to mechanical stress, facilitating microtubule self-repair mechanisms (Janke and Montagnac, 2017). It reduces inter-protofilament interactions and confers resilience against repeated mechanical stresses (Portran et al., 2017). Consistently, the depletion of the αK40 writer (the tubulin acetyltransferase TAT1) led to a significant increase in the frequency of microtubule breakage (Xu et al., 2017). Loss of this modification is associated with neuronal degeneration (Neumann and Hilliard, 2014). In the absence of K40 acetylation, microtubules in c3da neurons can be mechanically damaged (Yan et al., 2018).

Actin/spectrin rings also appear to be involved in the tolerance to mechanical stress. They act as load-bearing elements able to discharge the mechanical stress by the unfolding and re-folding of spectrin tetramers, working as “shock absorber” (Dubey et al., 2020). Krieg and colleagues showed in touch receptor neurons of *C. elegans* that more players could contribute to mechanical neuroprotection, with the actin-spectrin networks providing tension, MT bundles conferring stiffness, and microtubule-associate proteins like tau acting as dissipator of torsional forces (Krieg et al., 2017).

In summary, many elements could work in combination to confer resilience against mechanical stress to neurons for all our long life; the emerging understanding will yield important insights on aging and neurodegeneration studies.

## 7 The challenge of observing mechanical degradation

Uncovering the effects of repetitive stress on neurons necessitates high-resolution imaging techniques to visualize subcellular changes within the cytoskeleton. The cytoskeleton of neurons is tightly packed composed of nanometric elements, including microtubule individual fibers spaced as close as 50 nm apart (Yamada et al., 1971). The microtubule density can reach up to 150 fibers within just one μm square (Wortman et al., 2014). While it is clear that mechanical load affects the microtubules within a living cell as discussed in the previous section, the diffraction limit which is typically over 200 nm (Born and Wolf, 1986) prevents us from getting to the single microtubule fiber or even to a bundle resolution *in vitro* (Kabir et al., 2020). Therefore, imaging the details of the neuronal cytoskeleton requires advanced microscopy techniques that can bypass this limit (Leterrier et al., 2017; Werner et al., 2021).

**Figure 5:**
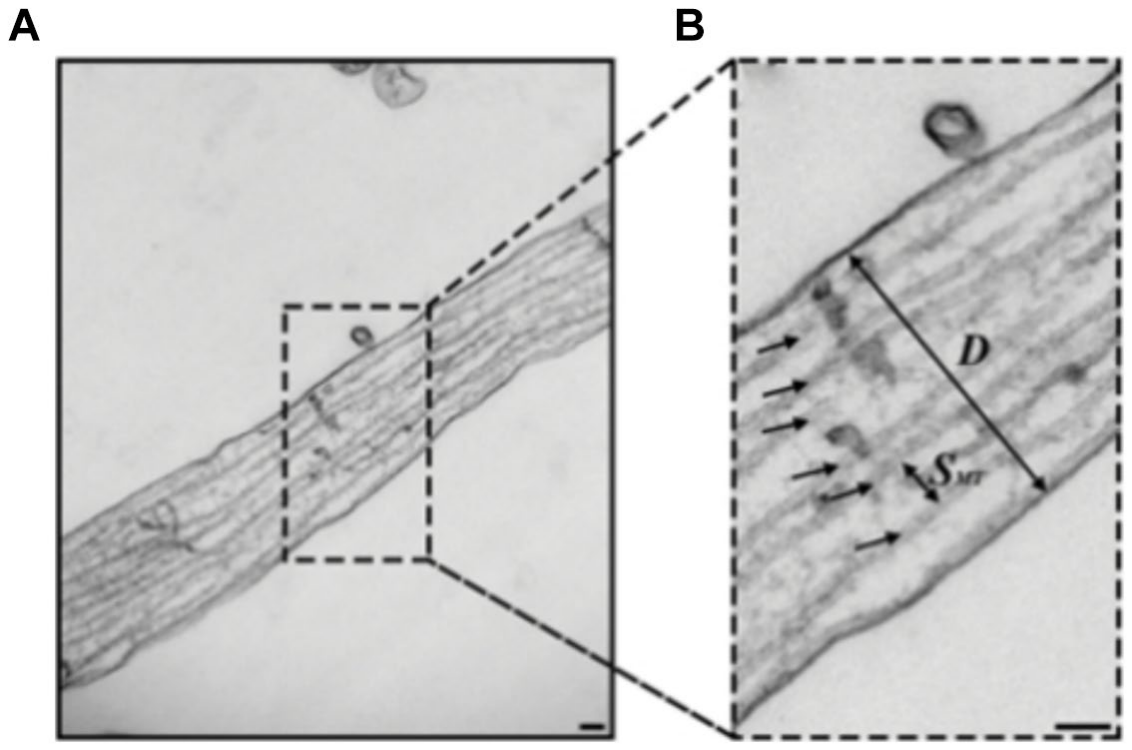
**(A,B)** Transmission electron microscopy (TEM) images of hippocampal rat neurons cell’s axon with microtubules indicated by black arrows **(B)**, Scale bar: 100 nm. Figure was adapted from Fournier et al. (2015).

While electron microscopy (EM) with its resolution of a few nanometers provides the capability to resolve individual microtubules as seen in Figure 5 (Lunn et al., 1997; Yang et al., 1999; Ventura Santos et al., 2023), it faces significant challenges when it comes to simultaneously imaging multiple targets as it involves heavy metal staining, which can affect sample integrity and hinder antibody binding to its target resulting in a poor contrast and inability to differentiate between different targets (Kim et al., 2020; Lelek et al., 2021). Moreover, it requires complicated sample preparation with harsh conditions which can introduce artifacts and are not suitable for live cells (Inkson, 2016). Therefore, super-resolution (SR) optical techniques that have been developed over the past two decades offer valuable complementary approaches, as they allow to overcome those challenges and open new possibilities for more comprehensive analysis.

**Figure 6:**
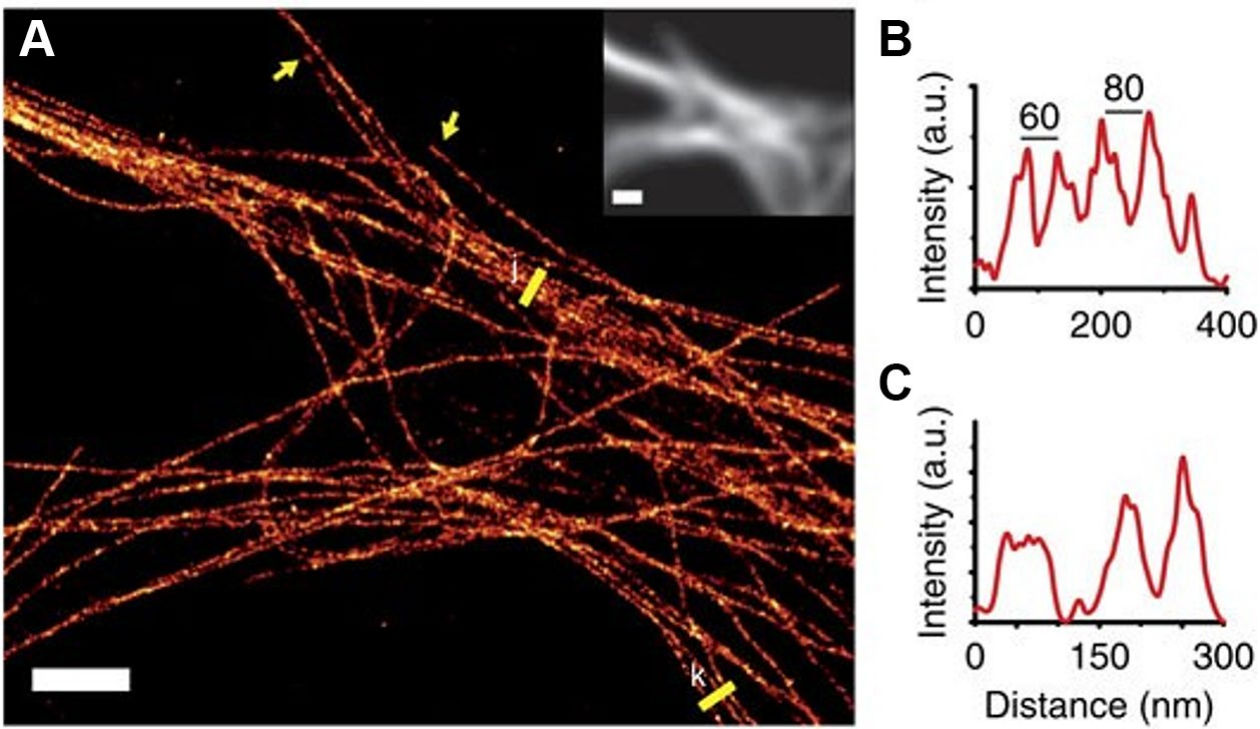
**(A)** SMLM reconstruction of microtubule bundles labeled with nanobodies in the dendrites of a hippocampal primary neuron. Yellow arrows indicate microtubule ends and yellow lines were used to create the profiles **(B,C)**. Inset shows the diffraction-limited fluorescence image. Scale bar, 2 μm. Figure was adapted from Mikhaylova et al. (2015).

Among the common SR techniques, single-molecule localization microscopy (SMLM) stands out due to its ability to achieve resolutions as fine as 10 nm (Rust et al., 2006), compared to 100 nm and 30 nm of Structured Illumination Microscopy (SIM) and Stimulated Emission Depletion microscopy STED, respectively, (Gustafsson, 2000; Willig et al., 2006), and were able to resolve the complex structure of the cytoskeleton, revealing actin rings (Xu et al., 2013; Papandréou and Leterrier, 2018) and single microtubule bundles and even fibers within the neurite, as can be seen in Figure 6 (Mikhaylova et al., 2015; Tas et al., 2017). Furthermore, some SMLM based techniques are compatible with live cells (Shroff et al., 2013; Zhang et al., 2013). These characteristics are fundamental when examining the characterization and dynamic behavior of a minute structure such as the neuronal cytoskeleton (Wang and Brown, 2002; Sakakibara et al., 2013). However, even though SMLM holds the theoretical capability to achieve the desired resolution, it is challenged by a densely packed structure such as the neuronal cytoskeleton. Images of these structures are often characterized by increased noise levels, resulting in diminished SNR and consequently inferior resolution. To overcome this challenge, certain modifications to the SMLM approach are necessary.

One approach that was explored in the last decade, is to combine SMLM with other advanced techniques, such as expansion microscopy (ExM) resulting in Ex-SMLM (Tong et al., 2016; Zwettler et al., 2020). This method increases the physical size of the sample and thereby mitigates its dense structure, enabling us to achieve a resolution like electron microscopy (Wang et al., 2014; Alon et al., 2021). ExM operates by introducing a hydrogel matrix to the sample and anchoring the target of interest, such as proteins, within the sample to the hydrogel. Subsequently, a chemical reaction, often as simple as water absorption, is employed to expand the hydrogel, leading to a uniform expansion of the entire sample (Chen et al., 2015; Alon et al., 2019; Gambarotto et al., 2021). Unfortunately, ExM must use fixed cells, and as a result does not allow the observation of dynamic events, such as damage processes resulting from mechanical stimulation and the ensuing repair mechanisms.

Measuring intracellular dynamics and integrity are crucial to comprehend cellular responses to mechanical stress that may serve as indicators of potential self-repair mechanisms (Schaedel et al., 2015; Théry and Blanchoin, 2021). One central factor in the characterization of these dynamics is the diffusion coefficient, a metric that quantifies the movement of particles across a predefined area. As mechanical stress progresses, any change in dynamics (e.g., diffusion coefficient) will indicate on the possible existence of self-repair mechanisms (Robert et al., 1990). Measuring these changes requires the determination of the diffusion coefficients of ensembles and individual particles and various microscopy techniques are employed to measure them. Fluorescent Recovery After Photobleaching (FRAP) enables us to access the diffusion coefficient for entire populations of molecules (Edson et al., 1993; Ishikawa-Ankerhold et al., 2012; Blumenthal et al., 2015). Single Particle Tracking (SPT) based techniques provide the means to measure several parameters on the single molecule level such as diffusion coefficient and directionality over time (Tinevez et al., 2017) even in densely packed areas by using photoactivatable dyes (Subach et al., 2010). Both approaches provide complementary information and improve our comprehension of cellular responses to mechanical stress.

## 8 Conclusion

The axons of peripheral nerves undoubtedly experience repetitive mechanical motion causing significant deformations. The effects of these deformations on the internal structure of an axon are still unknown, due to the difficulty of replicating the motion in cell culture and observing the dynamic changes with an imaging technique of sufficient resolution. While electron microscopy yielded detailed static images of the axon ultrastructure, we anticipate that the imaging of dynamic changes will require advances in optical imaging techniques. Continuous progress is being made in creating devices which allow the application of repetitive stresses to cells and cellular substructures, such as microtubules and microtubule bundles (Kabir et al., 2015, 2020; Abraham et al., 2018). However, the number of cycles is typically limited to hundreds rather than the 100 million deformation cycles experienced by a peripheral neuron in a human, which requires the extrapolation of the responses observed in such experiments (Abraham et al., 2018; Qiang et al., 2019). In the testing of engineered systems, “accelerated aging” where the stress level (e.g., by elevating temperature) is increased above the typically experienced values during testing has been proven to be of value (Hukins et al., 2008), and may be replicated here. Previous work on the response of cells to single and repeated mechanical stresses has highlighted to complexity of the cellular response, which depends on the specific nature of the deformation (e.g., tensile or compressive) and the precise time course of stress application. Often a threshold behavior is observed, where damage is detectable above a certain level of stress, and cell death occurs at an even higher level. An intriguing question is of course if mechanical stress can have a hormetic effect (Calabrese and Mattson, 2017). Despite the theoretical and experimental challenges associated with this research topic, new information about the mechanical response of cells and subcellular components is constantly being generated and contributes to the elucidation of the key mechanisms. Progress in our understanding of this topic promises clinical benefits related to diseases of aging as well as lessons for the bioinspired design of engineered structures.

## Author contributions

AC: Writing – original draft. AF: Writing – original draft. OM: Writing – original draft. MR: Writing – original draft. GS: Writing – original draft, Writing – review & editing. HH: Writing – original draft, Writing – review & editing. AK: Writing – original draft, Writing – review & editing. VR: Writing – original draft, Writing – review & editing. OS: Writing – original draft, Writing – review & editing. SRN: Writing – original draft.
